# Traumatic-event headaches

**DOI:** 10.1186/1471-2377-4-17

**Published:** 2004-10-29

**Authors:** David C Haas

**Affiliations:** 1 Department of Neurology, SUNY Upstate Medical University, University Health Care Center, 90 Presidential Plaza, Syracuse, NY 13202, USA

## Abstract

**Background:**

Chronic headaches from head trauma and whiplash injury are well-known and common, but chronic headaches from other sorts of physical traumas are not recognized.

**Methods:**

Specific information was obtained from the medical records of 15 consecutive patients with chronic headaches related to physically injurious traumatic events that did not include either head trauma or whiplash injury. The events and the physical injuries produced by them were noted. The headaches' development, characteristics, duration, frequency, and accompaniments were recorded, as were the patients' use of pain-alleviative drugs. From this latter information, the headaches were classified by the diagnostic criteria of the International Headache Society as though they were naturally-occurring headaches. The presence of other post-traumatic symptoms and litigation were also recorded.

**Results:**

The intervals between the events and the onset of the headaches resembled those between head traumas or whiplash injuries and their subsequent headaches. The headaches themselves were, as a group, similar to those after head trauma and whiplash injury. Thirteen of the patients had chronic tension-type headache, two had migraine. The sustained bodily injuries were trivial or unidentifiable in nine patients. Fabrication of symptoms for financial remuneration was not evident in these patients of whom seven were not even seeking payments of any kind.

**Conclusions:**

This study suggests that these hitherto unrecognized post-traumatic headaches constitute a class of headaches characterized by a relation to traumatic events affecting the body but not including head or whiplash traumas. The bodily injuries *per se *can be discounted as the cause of the headaches. So can fabrication of symptoms for financial remuneration. Altered mental states, not systematically evaluated here, were a possible cause of the headaches. The overall resemblance of these headaches to the headaches after head or whiplash traumas implies that these latter two headache types may likewise not be products of structural injuries.

## Background

Chronic headaches from head trauma [1-4] and whiplash injury [5-8] are well-known and common. Together with their accompanying symptoms they are usually referred to as "postconcussive (or post-traumatic) syndrome" and "whiplash syndrome," respectively. Chronic headaches from other sorts of physical traumas are not recognized, but a few authors have mentioned them [9-11]. Parker [9] reported that, among 750 consecutive litigants seen by him for industrial and motor-vehicle accidents, 53% of those who had sustained neither a head nor whiplash injury complained of headache. Duckro et al. [10] were perplexed about their patients with such headaches: "Even more puzzling, from a diagnostic standpoint, are those patients who suffer persistent exacerbation of headache following physical trauma not involving the head or neck." They also noted that "...in our experience at a university-based clinic for chronic head and neck pain, the problem is not uncommon." The present paper details a series of chronic headaches that began soon after and in apparent relation to various physical traumas that did not include head trauma or whiplash injury. It describes the traumatic events, their temporal relation to the headaches, the features of the headaches, and the symptoms associated with the headaches. It also analyses the relation of the headaches to the events and compares the headaches with those following head trauma and whiplash injuries.

## Methods

The 15 patients in this series were all those seen by the author from February 1997 through December 2001 for chronic headaches apparently related to traumatic events that involved the body but did not cause either head trauma or whiplash injury (a term denoting a painful cervical injury, typically occurring during a motor-vehicle collision and generally considered to be a cervical sprain) [7,12,13]. The patients themselves attributed their headaches to the events and so did most of their referring physicians. Detailed accounts of the events and the headaches had been obtained from the patients, family members sometimes, and medical records sent by the referring physicians. Before their events, none of the patients had more than minor occasional headaches.

Detailed information had been systematically collected on the headaches' characteristics (intensity, location, quality, and response to physical activities), duration and frequency (when episodic), and accompaniments (nausea, vomiting, hypersensitivity to light and noise), and on the patients' use of pain-alleviative drugs. With this information, the headaches were classified by the 1988 diagnostic criteria of the International Headache Society (IHS) [14], extant during this review, as though they were naturally-occurring headaches [15]. Information on the presence of other post-traumatic symptoms and litigation had also been recorded, but less systematically than that for the headaches.

## Results

### The physical traumas

The physical traumas were very diverse (Table 1). None occurred in motor-vehicle accidents. One patient had his face cut by a metal sign as he fell, but he was not stunned by this contact. Two patients lost consciousness briefly, one from syncope after giving blood, and the other from a high-voltage electrical current. Six events produced *identifiable *damage to a part of the body (patients 1, 2, 9, 10, 13, 15). All but two of the events (patients 8, 11) were sudden and unexpected.

**Table 1:** Patients with headaches from diverse traumatic events.

Patient	G/Age	Traumatic event	Headache onset	Headache class*
1	M/38	He slid down a roof into a wall and broke a foot and back bone.	4 weeks	2.2
2	F/50	A falling mass of snow buried her and fractured 3 back vertebrae.	2 weeks	2.2
3	F/36	She fainted after donating blood.	1 day	2.2
4	M/41	He was knocked into his car when another car hit his shopping cart.	Immediate	2.2
5	M/46	He hurt his neck while yanking a wrench on a rusted bolt.	5 days	2.2
6	M/52	He heard his neck "pop" while lifting a cargo door.	Hours	2.2
7	M/28	He slipped off a plank while carrying buckets but landed on his feet.	Days	2.2
8	M/43	He had a bone-marrow transplant for a lymphoma.	Days	2.2
9	M/48	His face was badly cut on a metal sign when he fell while walking.	Hours	2.2
10	F/56	Her scalp was burned by a hair-curling chemical at a beauty parlor.	1 day	1.1
11	M/40	He became angry at the urologist right after his cystoscopy.	1 hour	2.2
12	F/47	Her snowmobile was mistakenly backed up 5 feet into a tree.	Days	2.2
13	M/53	He fell sideways in a beachchair and fractured his ribs on a rock.	2 days	2.2
14	F/33	She was exposed to an acute Freon-gas leak at work.	Minutes	1.1
15	M/43	A high-voltage electrical injury led to amputations of both arms.	4 weeks	2.2

*IHS codes: 2.2 = chronic tension-type headache, 1.1 = migraine without aura

### Intervals between traumatic events and headaches

The reported intervals between the traumatic events and the headaches' onsets are listed in Table 1. These intervals can be placed into four groups: *within minutes *(patients 4, 14), *within hours *(patients 6, 9, 11), *within days *(patients 3, 5, 7, 8, 10, 12, 13), and *within weeks *(patients 1, 2, 15). The interval for the one patient who did not have an abrupt traumatic event (patient 8) was arbitrarily set as the interval between his hospital discharge and headache onset.

### The headaches

Thirteen of the 15 patients had serious continuous headaches of varying intensity. Among these, 11 patients had headaches that met the IHS's diagnostic criteria for *chronic tension-type headache *[14] fully, while the other 2 patients' headaches met all but one of the criteria. The 2 patients without continuous headaches had frequent headaches that met the criteria for *migraine without aura*, and one of them also had headaches fitting the criteria for *migraine with aura *[14]. Table 1 lists each patient's headache class.

After the completion of this study, the IHS added a new primary headache class called *new daily-persistent headache*, which has the same features as chronic tension-type headache, but is distinguished from it by becoming daily and unremitting within three days of its onset [16]. Many, if not all, of the 13 patients in this study with continuous headaches may have had their headaches develop in this way, but as information about this was not specifically sought and as this distinction is of unproven merit, *chronic tension-type headache *is used herein.

The IHS's criteria for chronic tension-type headache and migraine without aura [14] are listed below in abbreviated form.

#### Chronic tension-type headache

Headache frequency more than 15 days/month.

At least 2 of the following pain characteristics:

1. Pressing quality

2. Mild or moderate severity

3. Bilateral location

4. No aggravation by routine physical activity

Both of the following:

1. No vomiting

2. No more than one of the following: Nausea, photophobia or phonophobia

#### Migraine without aura

Headaches last 4 to 72 hours.

Headache has at least 2 of the following characteristics:

1. Unilateral location

2. Pulsating quality

3. Moderate or severe intensity

4. Aggravation by routine physical activity

During headache at least one of the following:

1. Nausea and/or vomiting

2. Photophobia and phonophobia

### Examples of chronic tension-type headache after traumatic events

#### Patient 5

This 46-year-old man was seen in 1999 for headache that began five days after an "injury" at work five months earlier. Ten minutes after forcefully yanking a wrench to loosen a rusted bolt, he felt pain in his neck and right shoulder. An urgent-care facility prescribed analgesics after taking (unremarkable) cervical radiographs. This pain disappeared before I saw him. The headache was a continuous dull to moderate ache mostly in the right cranium. It was unaffected by physical activities or neck movements, and was not nauseating. Neurological examinations were normal. Pressure on his posterolateral neck was not painful. A cranial CT was normal. His only other symptom was insomnia. Analgesics, taken just a few days per week, had little effect. Amitriptyline lessened the headache's intensity enough for him to return to work.

#### Patient 9

This 48-year-old man with a neurologic impairment of gait was seen in 1999 for continuous headache that began a few hours after he fell while walking two months earlier and cut his face on the edge of a metal sign. His head was not struck and he was not stunned. He bled profusely from the laceration, which was closed with 26 stitches in the emergency room. His neurologist detected no new neurologic findings and a cranial CT was normal. His headache was a non-nauseating steady ache of mild to moderate intensity in his forehead, unaffected by exercises, brightness, or noise. Non-prescription analgesics and opioids had been ineffective and discontinued.

#### Patient 11

This 40-year-old man was seen in 2000 for a continuous headache that began 10 months earlier soon after he awoke from anesthesia for a cystoscopy. When the urologist did not report the (negative) result of the procedure to him in the recovery suite, the patient became visibly angry and soon complained about his treatment to the health-care facility. His anger persisted and was expressed at his consultation. The headache was a steady non-nauseating pain that fluctuated from dull to moderate intensity at the vertex, temples, and posterior neck, and was unaffected by physical activities, brightness, or noise. He worked despite it and no longer took analgesics. A cranial MRI had been normal. A trial of amitriptyline had been unsuccessful. At his last report a month later he reported improvement on buspirone.

#### Patient 12

This 47-year-old woman was seen in 2001 for symptoms that she attributed to a snowmobile accident seven weeks earlier. She was seated behind the driver when he mistakenly shifted into reverse sending the machine backwards five feet into a tree. At impact, he fell on top of her without hurting her or himself. She recalled no impact of her helmet against the tree or the snowmobile. She felt no pain, but was upset and asked to be taken home. On the next day, her neck ached. Cervical radiographs taken eight days after the accident were normal. Two days later, she developed severe headache, nausea, dizziness, and confusion. A cranial CT taken later that day was normal. Subsequent MRIs of her head and neck were normal.

Headache soon became her most prominent pain. It was a continuous pressing ache of mild to moderate intensity in her temples and orbits, unaffected by physical activities. It was sometimes nauseating, without emesis. Brightness and noise bothered her. She also complained of dizziness and impaired thinking and memory. Infrequent doses of analgesics were not beneficial. She was unable to work. Neurologic examinations were normal. Preventive medications were refused. When seen next, by a colleague, two months after her visit with me, the headache and dizziness had lessened considerably, but her thinking difficulties remained disabling. Four months later, she reported continuing improvement.

#### Patient 13

This 53-year-old man was seen in 2001 for a headache of six-months duration that began two days after he struck the right side of his chest against a rock without striking his head when he toppled over in a beach chair. His chest pain was extreme. He obtained a prescription for hydrocodone/acetaminophen tablets that day, but discontinued taking them after several doses because of side effects. Coughing, sneezing, and lying on his right side were excruciating. His physician diagnosed a fractured rib. Two days after the injury, he returned to work despite his chest pain and new headache. The chest pain disappeared in a few weeks, but the headache persisted. It was a continuous, non-nauseating, bifrontal "tightness" of dull to moderate intensity, unaffected by mild physical activities, brightness or noise. Amitriptyline and propranolol had been ineffective and produced side effects. When I saw him, he was taking only occasional doses of non-prescription analgesics. Neurological examinations and cranial MRI were normal. He declined other medications. Seven months later, he reported that his headache persisted.

#### Patient 15

This 43-year-old man was seen in 2001 for a continuous headache that he first became aware of soon after discharge from hospital, in 1997, where he had undergone 25 days of intensive treatment for a high-voltage electrical injury that had necessitated amputation of his arms. Unconsciousness had been instantaneous, but brief, and post-traumatic amnesia lasted about ten minutes.

His headache was a bi-occipital, non-pressing ache, usually of mild to moderate intensity, and only occasionally severe enough to force him to cease physical activities. Then it was nauseating, without emesis. Some loud noises, but not brightness, seemed to intensify it. He had been getting slight relief from a few doses of ibuprofen per week, but had received no preventive medications.

His cognitive and emotional states and cranial MRI were unremarkable. He had been provided with prosthetic upper limbs with grasping hands. Amitriptyline decreased the headache slightly. The addition of progressively larger doses of dextroamphetamine limited the headache to only a few days per month.

### Other post-traumatic symptoms

Thirteen of the fifteen patients had, besides headache, at least one other post-traumatic symptom of the type commonly seen after head trauma [3,17,18]. Seven patients had either three or four symptoms. The number of patients having each of the symptoms is listed in Table 2. Only patient 2 had any of the symptoms included in the syndrome of post-traumatic stress disorder [19].

**Table 2:** Other post-traumatic symptoms.

Symptoms	Number of patients with symptom
Insomnia	14
Decreased concentration	7
Decreased memory	5
Dizziness	5
Mild depression	3
Anxiety	3
Tiredness	3

### Litigation/compensation

Seven patients (numbers 3, 5, 9, 10, 11, 12, 13) were not seeking and could not seek financial compensation for their headaches. Three patients (numbers 6, 7, 14) were receiving Workers' Compensation (WC) payments for their headaches and other symptoms. One patient (number 15) was receiving both WC and Social Security (SS) disability payments. One other receiving WC payments was also seeking SS disability payments (number 1). One patient (number 8) was seeking SS payments only. One patient (number 2) was litigating for payment of medical bills. Only one patient (number 4) had pursued a lawsuit for monetary compensation for the symptoms, but he continued to experience headaches even after receiving the award.

## Discussion

This report has presented evidence suggesting that traumatic accidents and other events without head trauma or whiplash injury but with other physical effects can induce chronic headaches. Such headaches have barely been alluded to before (see Background). The 15 patients presented here blamed their headaches and other symptoms on their traumas and are supported in this by the juxtaposition of their headaches to the traumas. Twelve of the 15 patients developed their headaches minutes, hours, or a few days afterwards (Table 1). These intervals are well within those deemed necessary by the IHS for connecting both head trauma and whiplash injury to subsequent headaches. Their 1988 classification [14] lists "less than 14 days" but their 2004 revision [16] lists "within 7 days" as the maximum interval acceptable for relating chronic headaches to these injuries. The other 3 patients reported headache onsets of two weeks, four weeks, and four weeks. Nevertheless, they are included in this series because the apparent connection of their headaches to their serious accidents outweighs the still non-evidential latent-period requirement chosen by the IHS. In support of this inclusion is evidence suggesting that genuine post-traumatic symptoms can develop as late as three months after head trauma [11,20] and that chronic headaches after whiplash injuries may not appear for weeks or even months [21].

Although the headaches in this study were temporally related to events involving the body, they were unlikely to have been caused by the bodily injuries themselves, for there is no conceptual link between injuries such as a facial laceration or a fractured rib and chronic headaches. Moreover, nine patients had either trivial or unidentifiable injuries (Table 1, patients 3, 4, 5, 6, 7, 8, 11, 12, 14). Fabrication of symptoms for financial remuneration was not evident in this series, in which seven patients were not even seeking payments of any kind. If these headaches can not be attributed to bodily injury, then the symptoms would appear to be of psychological origin, as they are after some other sorts of traumatic events, such as those that set off chronic "post-traumatic stress disorder" (PTSD) [19]. Traumatic psychological symptoms can include headache. It has been reported, for example, to accompany the characteristic symptoms of PTSD [22], and it was the most prominent post-traumatic symptom in a "mass psychogenic illness" induced by false perceptions of exposure to toxic fumes at a school [23]. In this study the psychological states of the patients were not investigated (though they were not ignored), because the study's purpose was to analyze the traumatic events, the headaches following them, and the relationship between the two. Thus, this study can not present positive evidence for a psychological basis of the headaches. The headaches were not, however, related to PTSD, since only one patient had (some) symptoms of this condition.

Both head-trauma and whiplash headaches form clinical classes based on their preceding traumas, but the headaches of the present series have no single trauma to join them. They were, however, all related to acute and unexpected (with one exception) traumatic events that affected the patients physically. Hence, they could be designated *traumatic-event headaches*. Recognition of this class of post-traumatic headaches would link heretofore puzzling individual phenomena (see Background) and thereby foster their investigation.

The headaches themselves were serious chronic headaches. They had the features of continuous chronic tension-type headaches in 13 patients and frequent migraines in the other two [14,16]. This distribution of headache types is similar to that of the chronic post-traumatic headaches after head-trauma and whiplash injury (excepting the advocated cervical syndrome from some whiplash injuries [24]). In studies using the 1988 IHS diagnostic criteria, head-trauma headaches were 75% chronic tension-type, 21% migraine, and 4% unclassifiable [15], whiplash headaches were 74% tension-type, 15% migraine, and 11% cervicogenic [6], and "cranio-cervical acceleration/deceleration trauma" headaches (22% with head trauma, 78% with whiplash) were 37% tension-type, 27% migraine, 18% cervicogenic, and 18% unclassifiable [25]. In addition to the headaches, the present series of patients suffered other symptoms like those in the post-concussion [3,17,18] and whiplash syndromes [5,26] (Table 2).

The apparent overall likeness of the traumatic-event headaches to those after head and whiplash traumas suggests a pathogenic link between them. Such a link would be discredited if, as many believe, headaches after head and whiplash traumas are due to structural injuries to the brain and neck, respectively [21,24,27-29]. But such injuries have not been seen in cranial or cervical MRIs in the great majority of cases and whatever injuries may be present have an uncertain relation to the chronic headaches [7,28,30,31]. Moreover, certain evidence seems incompatible with the presence of symptomatic injuries. Firstly, the incidence of chronic symptoms after head trauma [2,3,28] or car crashes [32,33] does not increase in step with increasing degrees of trauma. Secondly, the development of chronic symptoms is dependent on the circumstances in which the trauma occurs. For example, whereas chronic headaches are common after head traumas at work, they are rare after head blows during sports [34-36]. Likewise, chronic whiplash symptoms have not been produced in volunteers subjected to rear-end car crashes [31,33] and they do not occur in demolition-derby drivers [37] and drivers or passengers subjected to rear-end crashes in certain countries where whiplash is not common knowledge [8]. This and additional evidence has led some authors to discount a role for cranial or cervical injuries in the development of chronic post-traumatic symptoms, offering instead explanations based on altered mental states [2,8,31,38,39]. These include, among others, neurotic reactions and culturally-related expectations of symptom development. The present study supports a mental origin from another perspective by showing that chronic post-traumatic headaches can be sequelae of traumatic events having neither head trauma nor whiplash injury.

## Conclusions

This study indicates that chronic headaches similar to those after head trauma and whiplash injury can follow other types of acute and unexpected physically injurious traumatic events. The evidence suggests that these traumatic-event headaches are psychogenic. The occurrence of such headaches supports the concept that the chronic headaches after head trauma or whiplash injury may likewise not be products of structural injuries.

## Competing interests

The author(s) declare that they have no competing interests.

## Authors' contributions

DCH was the sole investigator and author.

## Pre-publication history

The pre-publication history for this paper can be accessed here:

http://www.biomedcentral.com/1471-2377/4/17/prepub
